# Roles of Coactivators in Hypoxic Induction of the Erythropoietin Gene

**DOI:** 10.1371/journal.pone.0010002

**Published:** 2010-04-02

**Authors:** Feng Wang, Ruixue Zhang, Xiaomeng Wu, Oliver Hankinson

**Affiliations:** 1 Department of Pathology and Laboratory Medicine, and Jonsson Comprehensive Cancer Center, University of California Los Angeles, Los Angeles, California, United States of America; 2 Department of Microbiology, Immunology and Molecular Genetics, University of California Los Angeles, Los Angeles, California, United States of America; National Institute on Aging (NIA), National Institutes of Health (NIH), United States of America

## Abstract

**Background:**

Hypoxia-inducible expression of the erythropoietin (EPO) gene is mediated principally by hypoxia-inducible factor 2α (HIF-2α) in Hep3B cells under physiologic conditions. How/whether p300/CBP and the members of p160 coactivator family potentiate hypoxic induction of endogenous EPO and other HIF-2α and hypoxia-inducible factor 1α (HIF-1α) target genes remains unclear.

**Methodology/Principal Findings:**

We demonstrate, using chromatin immunoprecipitation (ChIP) analysis, that the histone acetyl transferase (HAT) coactivators p300, SRC-1 and SRC-3 are recruited to the 3′ enhancer of the EPO gene upon hypoxic stimulation, and that each associates with the enhancer in a periodic fashion. Hypoxia induced acetylation of the EPO gene 5′ promoter at histone 4 and lysine 23 of histone 3. Knocking down SRC-3, but not SRC-1 or SRC-2, using short interfering RNAs (siRNAs), reduced EPO transcriptional activity. Knocking down p300 resulted in dramatic down-regulation of hypoxic stimulation of EPO gene transcription, negated recruitment of RNA polymerase II to the gene's promoter, and eliminated hypoxia-stimulated acetylation at the promoter and recruitments of SRC-1 and SRC-3 to the enhancer. The inhibitory effects of knocking down p300 and the chromatin remodeling coactivator, Brm/Brg-1, on EPO transcription were additive, suggesting that p300 and Brm/Brg-1 act independently. p300 was also required for hypoxia induced transcription of the HIF-1α target gene, VEGF, but was dispensable for induction of two other HIF-1α target genes, PGK and LDHA. Knocking down CBP, a homolog of p300, augmented hypoxic induction of VEGF, LDHA and PGK. Different HIF target genes also exhibited different requirements for members of the p160 coactivator family.

**Conclusions/Significance:**

p300 plays a central coactivator role in hypoxic induction of EPO. The coactivators exhibit different specificities for different HIF target genes and each can behave differently in transcriptional regulation of different target genes mediated by the same transcription factor.

## Introduction

Tissue oxygen concentration is an important regulatory stimulus for many physiological and pathological processes [1], [2]. Adaptation to hypoxia depends in part on appropriate alterations in the expression of a number of physiologically relevant genes. Induction of the erythropoietin (EPO) gene by hypoxia is central to the regulation of the oxygen-carrying capacity of the blood [3]. Cellular adaptation to hypoxia is mediated in large part by the transcriptional activation of genes by Hypoxia-inducible Factor (HIF). HIFs are heterodimeric proteins containing one α subunit and one β subunit. HIF-1α and HIF-2α (collectively called the HIF-α subunits) are both expressed widely, as is HIF-1β (also called the Aryl Hydrocarbon Receptor Nuclear Translocator {ARNT}), while HIF-2β has a more limited tissue distribution. Under normoxic conditions, the HIF-α subunits are hydroxylated on key proline residues located in the C-terminal half of the proteins by O_2_–dependent prolyl hydroxylases, which allows for their recognition by the von Hippel-Lindau (pVHL) tumor suppressor protein that targets HIF-α for proteosomal degradation. Another level of HIF-1α inhibition occurs through the hydroxylation of an asparagine residue, also located towards the C-terminus of the protein, by the O_2_ -dependent Factor Inhibiting Hypoxia Inducible Factor-1α (FIH). This hydroxylation prevents HIF-1α from interacting with the coactivator p300 under normoxic conditions [4]. During hypoxia, HIF-1α dimerizes with its partner ARNT, and this complex then binds hypoxia response elements (HREs) in the promoter regions of target genes and up-regulates their transcription. Expression profiling and functional studies have revealed that the HIF-α subunits regulate both shared and unique target genes. Domain-swapping and chromatin immunoprecipitation studies have shown that selective HIF target gene activation is not based on the DNA-binding properties of these factors, since both HIF-α subunits can bind to the endogenous HREs of hypoxia-responsive genes, but that selectivity resides in their C-terminal regions, harboring their transcriptional activation domains.

The human EPO gene has been a powerful tool for studying the regulation of gene expression by HIF, since that the hypoxic induction of EPO expression is the most robust among hypoxia-inducible genes, and because the enhancer and promoter are located in 3′ and 5′ flanking regions of the gene, respectively, and widely separated (approximately 3 kb) from each other. Transcriptional regulation of the EPO gene is achieved by the concerted action of several transacting factors interacting with the proximal promoter region and with the 3′ untranslated enhancer region of the gene [5]–[11].

In eukaryotes, the regulation of transcription initiation requires that transcription factors function in the context of chromatin. Several classes of chromatin remodeling enzymes have been identified that facilitate transcription from the chromatin template, including histone acetyltransferases (HATs) and ATP-dependent remodeling enzymes [12]. p300 and its homolog, the CREB-binding protein (CBP), possess intrinsic HAT activity. These coactivators bind a number of sequence-specific transcriptional activators and have been suggested to be central integrators of transcriptional signals from various signals transduction pathways [13]. They have been implicated in HIF-mediated transcriptional activation of hypoxia-inducible genes via direct interactions with HIF-1α [14], [15], [16], [17]. The p160 steroid receptor coactivator (SRC) gene family contains three homologous members, SRC-1 (NCoA-1), SRC-2 (GRIP1, TIF2, or NCoA-2) and SRC-3 (p/CIP, RAC3, ACTR, AIB1, or TRAM-1), which serve as transcriptional coactivators for nuclear receptors and certain other transcription factors [18]. They possess many features in common and may be able to partially compensate for each other's functions [19], [20], [21]. SRC-1 and SRC–3 also possesses intrinsic HAT activity, though the HAT activities of SRC-1 and SRC–3 are much weaker than those of p300 and CBP [22], [23]. SRC-1 and SRC–2 have been shown to be able to interact with HIF-1α and to enhance its transactivation potential in a hypoxia-dependent manner, and this interaction is mediated by CBP [14], [15]. However, these findings are based on protein-protein binding, co-localization and reporter assays. It remains unclear how these coactivators contribute to the transcription of endogenous hypoxic-inducible genes and to histone modification at the promoters of these genes.

In this study, we investigated the roles of p300 and p160 family members in transcriptional regulation of the EPO gene and in chromatin modification at the native promoter region of the gene. We also investigated the relationship among p300, the p160 family members and the Brm/Brg-1 ATPase subunits of the mammalian SWI/SNF chromatin-remodeling complex in transcriptional regulation of the EPO gene and other HIF target genes.

## Methods

### Cell Culture and antibodies

The Hep3B cell line was purchased from ATCC and grown in modified Eagle medium (ATCC) with 10% fetal calf serum (Omega), L-glutamine (Invitrogen), fungizone, and penicillin-streptomycin (Invitrogen) at 37°C and 5% CO_2_. The Brm (N-19), Brg-1 (H-88), p300 (N-15), SRC-1 (M-341), and SRC-3 (C-20) antibodies were purchased from Santa Cruz Biotechnology, and the SRC-2 antibody from BD Biosciences were used for the both ChIP and western Blot assays, except that the SRC-1 (128E7) antibody from Cell Signaling, and the SRC-3 (1A8) antibody from Santa Cruz were used for western blot analysis. The Antibodies to Ac-K5, K8, K12, K18-H4, Ac-K14-H3, and Ac-K23-H3 were purchased from Upstate Biotechnology. The affinity-purified rabbit polyclonal antibody to ARNT was described previously [24].

### RNA interference in Hep3B cells

The pQCXIPgfp vector (a kind gift from Dr. Stephen Smale, UCLA), was modified for retroviral delivery of RNAi. The mouse U6 promoter was cloned into the NheI site within the 3′LTR [25]. Oligonucleotides encoding siRNA hairpins against p300 and SRC-2 were then annealed and cloned downstream of the U6 promoter. The targeting sequences for p300 and SRC-2 were 5′-CCCCTCCTCTTCAGCACCA-3′ and 5′-AGAGCAAACTCATCCGTTC-3′, respectively. For retrovirus generation, 293T cells were purchase from ATCC and grown in DMEM with 10% FBS and penicillin/streptomycin. Cells were grown to 90% confluency in 10-cm dishes and transfected with the retroviral vectors and the 10A1 packaging vector using Lipofectamine 2000 (Invitrogen). The medium was replaced 24 h post-transfection. The virus supernatants were collected 36 and 48 h after transfection, filtered through a 0.45-µm syringe filter, and stored at −80°C. For retroviral infection, Hep3B cells (3×10^5^/well) were seeded in six-well plates, and 2 mL of virus supernatant supplemented with polybrene (8 µg/mL final concentration) was added to the cells. Spin infections were performed at 2500 rpm for 1.5 h at 30°C. One day after the second infection, puromycin (3 µg/mL) selection was started and GFP expression was monitored by flow cytometry. RNA oligonucleotides were used for simultaneous RNAi knockdown of both Brm and Brg-1 (siB/B) [26], and for SRC-1 and SRC-3. The siRNA oligonucleotides were synthesized by Qiagen and annealed according to the manufacturer's protocol. The sense sequences of siSRC-1, siSRC-3, siB/B, siCBP and SCX, a scrambled RNA oligonucleotide, are r(CUCCUAAUAUUUCGACAUUAA)d(TT), r(CAGGAUUAUAUGGACAGACAU)d(TT), r(GCUGGAGAAGCAGCAGAAG)d(TT), r(GCACAGCCGTTTACCAUGAUU) and r(UUCUCCGAACGUGUCACGU)d(TT). Hep3B cells cultured in 6-well plates were transfected at 30% cell confluency using the Oligofectamine transfection reagent (Invitrogen). Western blot was performed to monitor degree of knockdown of the corresponding proteins.

### Reverse Transcription and Real-time PCR

Cells transfected with siRNAs were harvested forty-eight hours after transfection. Cells stably expressing the short hairpin RNAs were harvested after selection with puromycin. For measuring the mRNA of EPO, the plates were exposed to hypoxia 48 h after transfection and cultured for a further 24 h. Total RNA was prepared from transfected or infected cells using the RNeasy Micro kit (Qiagen). 1.5 µg of total RNA was reverse-transcribed using a Super script III Reverse Transcriptase kit (Invitrogen). The RT Real-Time SYBR/ROX PCR Master Mix was purchased from SuperArray; and PCR analysis was performed on an Applied Biosystems 7500 Real-Time PCR System, according to the instructions of the manufacturer. The primer sets for RT-PCR for the ribosomal 36B4, EPO and VEGF gene were as previously described (26). The primer sets for RT-PCR for LDHA and PGK are 5′-AAGTGGTTGCAATCTGGATTCAG-3′ and 5′-GGTGAACTCCCAGCCTTTCC-3′; 5′-CGCTTTCATGTGGAGGAAGAA-3′ and 5′- TGGCTCGGCTTTAACCTTGT-3′, respectively. The mRNA levels were normalized to that of the 36B4. Each of the real-time PCRs was done three times and one representative result is presented in each case. The result is an average from three real-time PCR reactions with the same template.

### Chromatin Immunoprecipitation Assay

The procedure was performed using a kit purchased from Upstate Biotechnology, according to the protocol recommended by the manufacturer. Briefly, Hep3B cells were treated with hypoxia or 100 µM desferrioxamine (DFO), for the indicated times. Cross-linking was achieved by adding formaldehyde to a final concentration of 1% at 37°C for 10 min. Cells were washed twice with ice-cold phosphate-buffered saline and collected in 1 ml of ice-cold phosphate-buffered saline. Cells were pelleted at 700×*g* at 4°C and resuspended in 0.3 ml of cell lysis buffer (50 mM Tris-HCl (pH 8.1), 10 mM EDTA, 1% SDS, Roche Complete protease inhibitor mixture), and incubated on ice for 10 min. Cell lysates were sonicated to give a DNA size range from 200 to 900 bp. Samples were centrifuged for 10 min at 4°C. Supernatants were diluted 10-fold with dilution buffer (16.7 mM Tris-HCl, pH 8.1, 1.1% Triton X-100, 1.2 mM EDTA, 167 mM NaCl, 0.01% SDS, and Complete protease inhibitor mixture). The solutions were pre-cleared with 80 µl of salmon sperm DNA/protein A agarose slurry for 30 min at 4°C, and then treated with antibodies overnight at 4°C. Immune complexes were collected using 60 µl of a salmon sperm DNA/protein A agarose slurry or protein G agarose (Amersham Pharmacia Biotec). The beads were pelleted and washed sequentially in the following buffers: low salt wash buffer (20 mM Tris-HCl (pH 8.1), 150 mM NaCl, 2 mM EDTA, 0.1% SDS, 1% Triton X-100); high salt wash buffer (20 mM Tris-HCl (pH 8.1), 500 mM NaCl, 2 mM EDTA, 0.1% SDS, 1% Triton X-100), LiCl wash buffer (10 mM Tris-HCl (pH 8.1), 0.25 M LiCl, 1% Nonidet P-40, 1% deoxycholate, 1 mM EDTA), and TE buffer (twice). Immuno-complexes were extracted from the beads with 1% SDS-0.1 M NaHCO_3_. Cross-linking was reversed by heating the eluates at 65°C overnight. The eluates were then digested with proteinase K at 45°C for 1 h. DNA was purified using a DNA purification kit (Qiagen). The 3′ enhancer region (+39 to +295 from the 3′ end of exon 5), and the 5′ promoter region (−368 to +18 from the transcription start site) of the EPO gene were amplified by PCR. The primer sequences for the enhancer and promoter of the EPO gene were 5′-GCCCTACGTGCTGTCTCACAC-3′ and 5′-CCTTGATGACAATCTCAGCGC -3′; 5′-ATGACCCACACGCACGTCTGCAGCAG-3′ and 5′-CGGGGCTGTTATCTGCATGTGTGCGT-3′, respectively. For real-time PCR analysis, the primer sequences for the enhancer and promoter were 5′- GCCCTACGTGCTGTCTCACAC -3′ and 5′-ATTGACCAGCGTAGGCAGAGC -3′; 5′-TGGCGACCCCTCACGCACACAGCC -3′ and 5′-TGGCCCAGGGACTCTGCGGCTCTGG -3′, respectively. Teal time PCR was performed as described in quantifying mRNA level. The DNA levels were normalized to those of the appropriate chromatin inputs after they are subtracted with DNA signals given by ChIP using normal rabbit or goat IgG. Each real-time PCR analysis was performed three times on the same ChIP and a representative result is presented. The result is an average from three real-time PCR reactions with the same template, unless specifically noted. Student's t test was used to determine the significances of differences between samples.

## Results

### Coactivator requirements during hypoxic induction of EPO transcription

**Figure 1:**
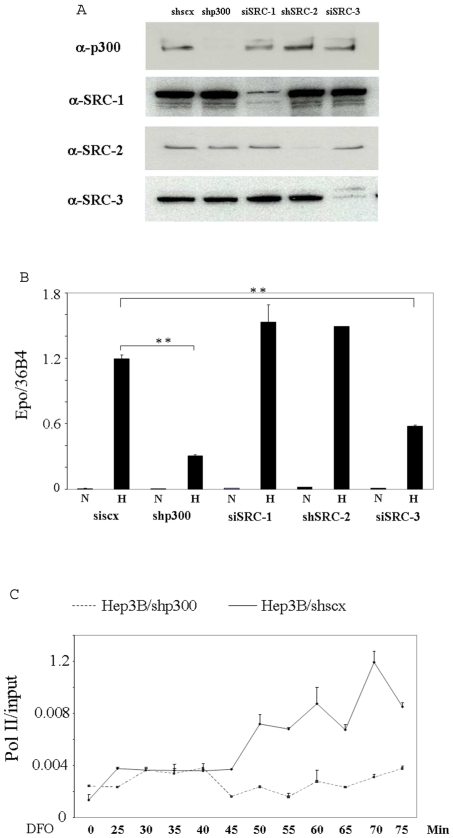
Requirements for coactivators in induction of the EPO gene by hypoxia. A. whole cell extracts were prepared from Hep3B/shscx, Hep3B/shp300, Hep3B/shSRC-2 and Hep3B cells transfected with siSRC-1 and siSRC-3 RNA duplexes for 72 h and then subjected to western blot analysis using the indicated antibodies. B, for analysis of EPO mRNA expression, the cells were treated with 1% O_2_ for 24 h, (Hep3B cells were transfected with siSRC-1 and siSRC-3 and 48 h later were treated with 1% O_2_ for additional 24 h). Total RNA was then isolated and subjected to reverse transcription and real-time PCR. EPO mRNA levels were normalized to the levels of the constitutively expressed 36B4 gene, encoding a ribosomal subunit. ** Indicates statistically significant difference (p<0.01). C, Hep3B/shscx and Hep3B/shp300 cells were treated with 100 µM DFO, harvested at the indicated time points, and subjected to ChIP analysis using an antibody directed to pol II. The precipitated chromatin was quantified by real time PCR using a pair of primers flanking the promoter of the EPO gene.

p300 has been shown to be required for the hypoxic induction of a reporter gene driven by the enhancer of the EPO gene, to associate with HIF-1α *in vitro*, and to form a complex with the HRE and HIF-1α under hypoxic conditions in a gel shift assay [16]. Furthermore, using transient transfected EPO enhancer driven reporter gene assays, immunoprecipitation assays and colocalization assays, two research groups found that SRC-1, SRC-2 and CBP, a homolog of p300, bind to HIF-1α and potentiate its transcriptional activity [14], [15]. We thought it important to extend these studies by investigating the roles of these coactivators in hypoxia induction of the endogenous EPO gene in its normal chromosomal configuration. Moreover, it should be noted that the hypoxic induction of EPO is mainly mediated by HIF-2α, rather than HIF-1α, in Hep3B cells under physiological conditions, although these cells express both HIF-α forms [27], [28]. The expression of EPO has also been shown to be dominantly regulated by HIF-2α in the adult mouse [28]. In order to examine the roles played by coactivators in hypoxia-induced transcription of the EPO gene, we knocked down individual coactivators using RNA interference. p300, SRC-1, SRC-2 and SRC-3 were knocked down in Hep3B cells by short hair-pin siRNA or siRNA oligonucleotides (Fig. 1A). While depletion of p300 and SRC-3 resulted in a significant reduction in transcription of the endogenous EPO gene triggered by hypoxia, depletion of neither SRC-1 nor SRC-2 inhibited the induced expression of the gene, suggesting either a redundancy of these p160 coactivators, or a dispensable role for both SRC-1 and SRC-2 in the transcription of the gene (Fig. 1B). We further found that desferrioxamine (DFO), a hypoxia mimicking reagent, stimulated a persistent association of RNA polymerase II (pol II) with the promoter in Hep3B/shSCX cells, which express a scrambled sequence with a short hairpin structure, but not in Hep3B/shp300 cells expressing a short hairpin RNA targeting p300, suggesting that p300 is required for the hypoxia induced association of pol II with the promoter (Fig. 1C). To exclude the possibility that the inhibitory effects of sip300 and siSRC-3 on EPO result from off target effects, we confirmed these results using siRNAs with different target sequences (Fig. S1 of Supplementary Information). We also thought it is important to confirm the lack of effect of depleting SRC-1 on the transcription of the EPO gene using another siRNA with a different inhibitory squence (Fig. S1 of Supplementary Information), since SRC-1 has been reported to play a role in the transcription of HIF-1α target genes in several independent studies using reporter gene assays.

### Coactivator Recruitment to the 3′ Enhancer Region of the EPO gene in Response to Hypoxic Stimulation

**Figure 2:**
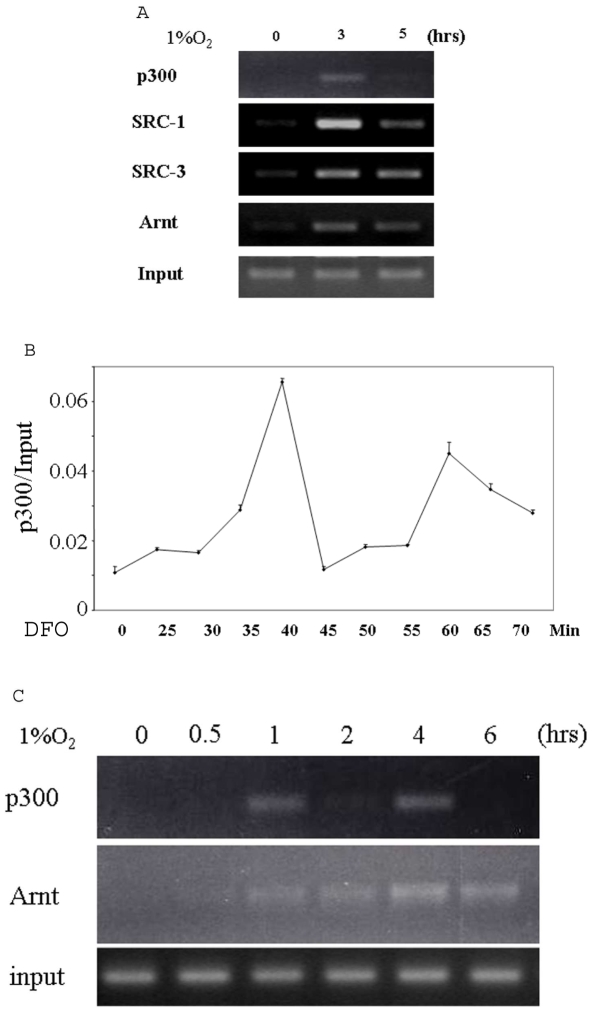
Coactivators are recruited to the EPO promoter upon hypoxic induction. A, Hep3B cells cultured under 21% O_2_ or 1% O_2_ for 3 h and 5 h were cross-linked and subjected to sonication and immunoprecipitation with the indicated antibodies. The precipitated chromatin was amplified by PCR using the primers flanking the enhancer of the EPO gene. B, Hep3B cells were treated with 100 µM DFO, harvested at the indicated time points, and subjected to ChIP analysis using an antibody directed to p300. The precipitated chromatin was quantified by real time PCR using a pair of primers flanking the enhancer of the EPO gene. C, ChIP was performed as described in A, except that the cells were harvested at different time points and only antibodies for Arnt and p300 were used.

We then performed ChIP analysis to test whether the coactivators are recruited to the 3′ enhancer region of the gene after hypoxia treatment. Antibodies against Arnt (HIF-1β), a common binding partner for both HIF-1α and HIF-2α, p300, SRC-1, and SRC-3 efficiently precipitated the 3′ enhancer region of the EPO gene in a hypoxia-dependent fashion (Fig. 2A), indicating that each associates with this region in response to hypoxic induction. p300 appeared to dissociate from the enhancer at the 5-hour time point, implying that it may bind to the enhancer in a cyclical fashion. Different coactivator complexes are recruited with distinct kinetics to target promoters by the corresponding transcription activators, as is evident from a number of well studied nuclear receptor systems [29], [30], [31]. Little is known about the kinetics of coactivator recruitment to the promoters or enhancers of hypoxia-inducible genes mediated by HIF. We therefore carried out kinetic ChIP studies on the 3′ enhancer region of EPO using an antibody against p300 in Hep3B cells. It was not feasible to use hypoxia for these experiments because the oxygen concentration fluctuates each time the incubator is opened, and it also takes a relatively long time for oxygen to equilibrate in the culture medium. We therefore used DFO. As shown in Fig. 2B, after quantifing the DNA obtained from ChIP for p300 using real time PCR, we found that p300 bound to the enhancer as quickly as 25 min after the addition of DFO, and associated with the enhancer in a periodic fashion. To confirm that this periodicity is not particular to DFO, we investigated the kinetics of p300 binding to the enhancer in cells cultured under hypoxia, using a time course with prolonged intervals between each time point. Again, we observed that p300 bound to the enhancer in a periodic fashion, while Arnt bound to the DNA stably and persistently (Fig. 2C), indicating that the periodic fashion of p300 recruitment does not result from a periodic association of the HIF-2α/Arnt transcription factor with the enhancer of the gene. We failed to detect any hypoxia induced binding of SRC-2 to the enhancer (data not shown), which is consistent with our finding that a siRNA to SRC-2 did not inhibit hypoxia induction of EPO.

### p300 is required for the recruitment of SRC-1 and SRC-3 to the enhancer of the EPO gene

**Figure 3:**
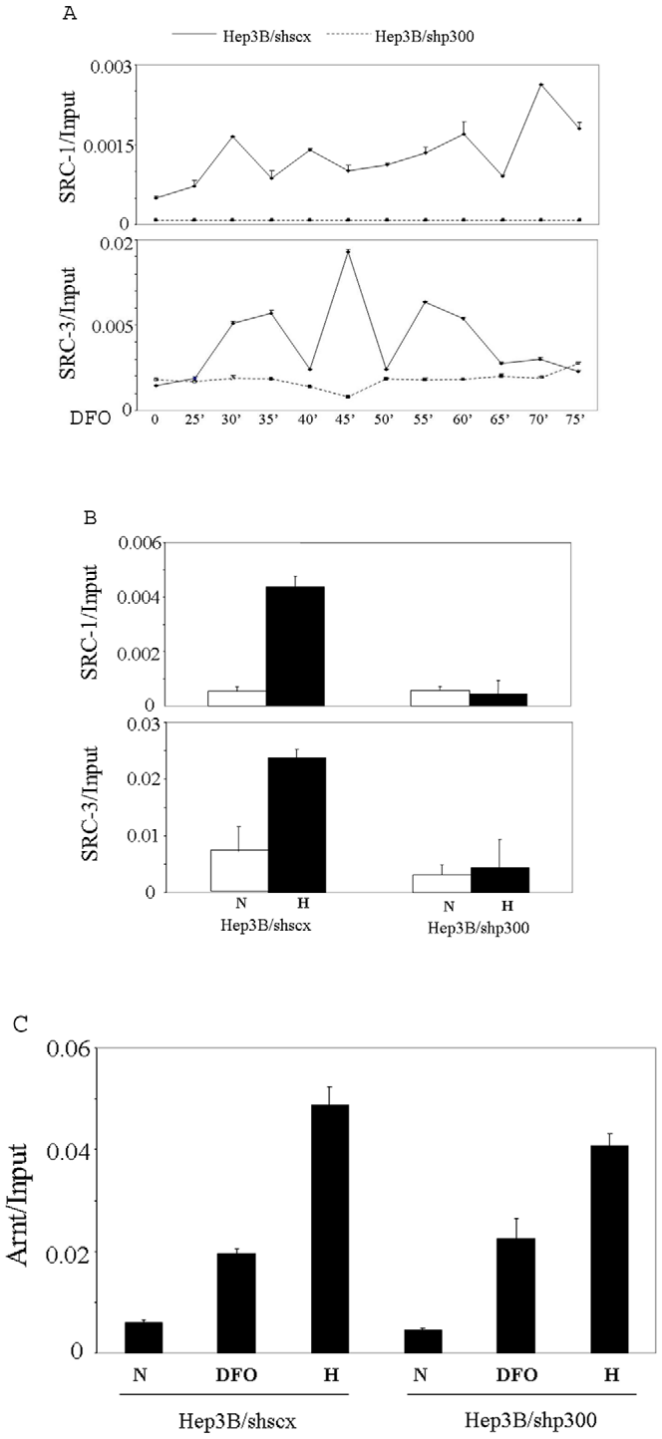
Hypoxia induced recruitments of the p160 coactivators are dependent on p300. A, Hep3B/shscx and Hep3B/shp300 cells were treated with 100 µM DFO, harvested at the indicated time points, and subjected to ChIP analysis using antibodies directed to SRC-1 or SRC-3. The precipitated chromatin was quantified by real time PCR. B, Hep3B/shscx and Hep3B/shp300 cells were treated with 1% O_2_ for 3 h and then subjected to ChIP analysis using antibodies against SRC-1 or SRC-3. The DNA obtained was quantified using real time PCR. C, Hep3B/shscx and Hep3B/shp300 cells were treated with 100 µM DFO for 1 h or 1% O_2_ for 3 h and then subjected to ChIP analysis using an antibody against Arnt. The precipitated chromatin was quantified by real time PCR.

In gene regulation mediated by nuclear hormone receptors, the p160 coactivators play major roles in chromatin remodeling and the assembly of general transcription factors through direct and indirect recruitments of other coactivators, including CBP/p300, although additional direct interactions between CBP/p300 and nuclear hormone receptors have also been observed [32], [18]. The p160 coactivators and CBP/p300 are also able to enhance the transactivition potential of HIF-1α in transient reporter gene assays, but in contrast to the above situation with the nuclear hormone receptor, CBP is required for SRC-1 to interact with HIF-1α, suggesting a major role for CBP in HIF-1α mediated gene regulation [14], [15]. We therefore examined the relationship of p300 to other coactivators during hypoxic induction of the EPO gene. We first investigated whether p300 impacts the recruitment of other coactivators to the enhancer of EPO. SRC-1 and SRC-3 bound to the 3′ enhancer region of EPO as quickly as 25 min after the addition of the hypoxia mimetic DFO to Hep3B/shscx cells, and each bound to the enhancer in a cyclic fashion. When we performed the same analysis in Hep3B/shp300 cells treated with DFO, we could not detect recruitment of either SRC-1 or SRC-3 to the enhancer of the gene throughout the whole time course, demonstrating that the recruitment of both SRC-1 and SRC-3 are dependent on p300 (Fig. 3A). Similarly, the hypoxia-stimulated recruitment of both SRC-1 and SRC-3 to the EPO enhancer was eliminated in cells knocked down for p300 (Fig. 3B). In contrast, recruitment of Arnt to the enhancer was unimpeded in Hep3B/shp300 cells either cultured under hypoxia or treated with DFO (Fig. 3C).

### Relationship between p300 and Brm/Brg-1

**Figure 4:**
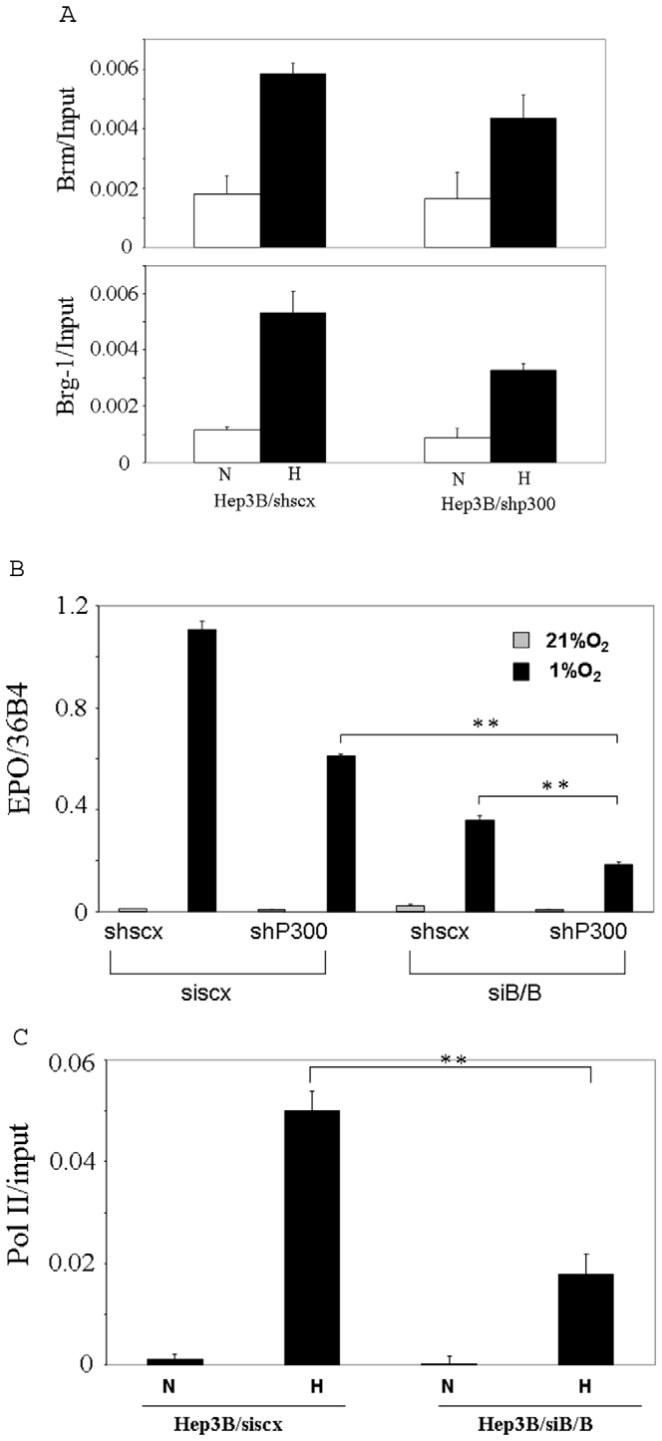
The relationship between p300 and Brm/Brg-1. A, Hep3B/shscx and Hep3B/shp300 cells were treated with 1% O_2_ for 3 h and subjected to ChIP analysis using antibodies against Brm or Brg-1. The precipitated chromatin was quantified by real time PCR. B, Hep3B/shscx and Hep3B/shp300 cells were transfected with siscx or siB/B. 48 h after transfection, the cells were treated with 1% O_2_ for 24 h. Total RNA were then isolated and subjected to reverse transcription and real-time PCR. C, Hep3B cells were transfected with siscx or siB/B. 72 h after transfection, the cells treated with 1% O_2_ for 3.5 h and then subjected to ChIP analysis using an antibody for RNA polymerase II. The precipitated chromatin was quantified by real time PCR. ** Indicates statistically significant difference (p<0.01).

We previously showed that Brm and Brg-1 are recruited to the enhancer of the EPO gene and that their diminution reduces induction of the EPO gene by hypoxia [26]. In this study, we investigated whether p300 affects hypoxia- stimulated recruitment of Brm/Brg-1 to the enhancer. As shown in Fig. 4A, knocking down p300 did not significantly impair the hypoxia-induced recruitment of either Brm or Brg-1 to the enhancer, suggesting that p300 and Brm/Brg-1 belong to different protein complexes. Hypoxic induction of EPO in cells with triple knock down of p300, Brm and Brg-1 was lower than that in cells with only p300 knocked down or in cells with only Brm/Brg-1 knocked down (Fig. 4B), further suggesting that p300 and Brm/Brg-1 belong to different protein complexes. Using ChIP analysis, we also found that simultaneously knocking down Brm and Brg-1 inhibited the hypoxia-stimulated recruitment of RNA polymerase II (pol II) to the EPO promoter, indicating that the SWI/SNF chromatin remodeling complex is required for the hypoxia induced association of pol II with the promoter (Fig. 4C).

### P300 is required for acetylation of histone at the EPO promoter associated with transcriptional induction of the EPO gene

**Figure 5:**
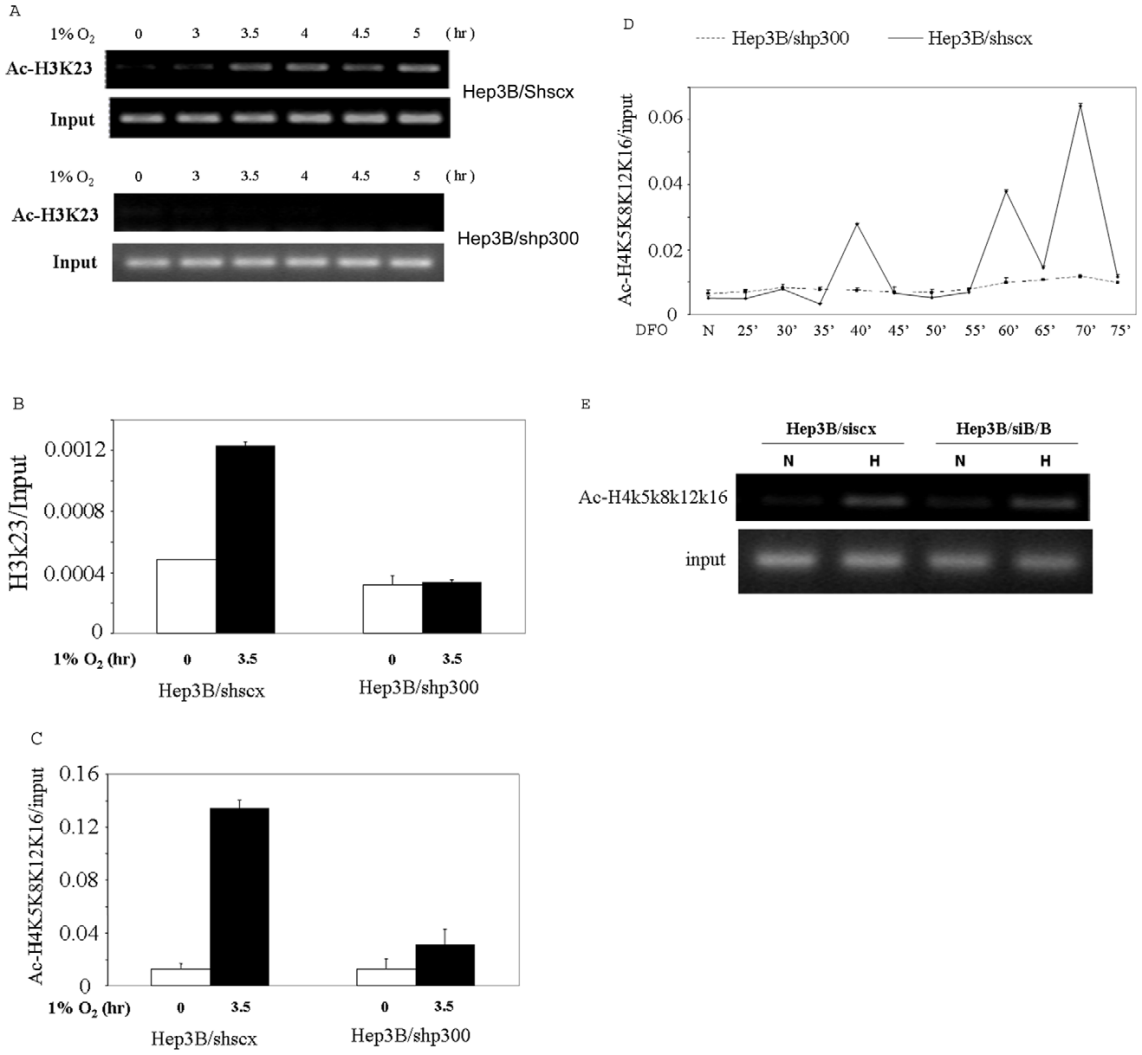
p300 is required for hypoxia-stimulated histone modifications at the EPO promoter. A, Hep3B/shscx and Hep3B/shp300 cells were cultured under 1% O_2_, harvested at the indicated time points, and subjected to ChIP analysis using an antibody directed to acetylated H3K23. B, DNA obtained from the ChIP described in A was quantified by real time PCR using a pair of primers spanning the promoter of the EPO gene. C, Hep3B/shscx and Hep3B/shp300 cells were cultured under 1% O_2_, harvested at 0 and 3.5 h, and subjected to ChIP analysis using antibodies directed to acetylated H4K5K8K12K16. The precipitated chromatin was quantified by real time PCR. D, Hep3B/shscx and Hep3B/shp300 cells were treated with 100 µM DFO, harvested at the indicated time points, and subjected to ChIP analysis using an antibody directed to acetylated H4K5K8K12K16. The precipitated chromatin was quantified by real time PCR. E, Hep3B cells were transfected with siscx or siB/B. 72 h after transfection, the cells treated with 1% O_2_ for 3.5 h or untreated (0 h) and then subjected to ChIP analysis using an antibody for acetylated H4K5K8K12K16.

Given that coactivators with intrinsic HAT activity, p300, SRC-1 and SRC-3, were recruited to the enhancer of the EPO gene in response to hypoxic stimulation, we speculated that, via a DNA-looping mechanism, the HIF/coactivator complexes modify histones at the 5′ promoter region, so as to make it become permissive for the assembly of an active preinitiation complex. We therefore analyzed histone modifications at the promoter region in Hep3B/shscx cells, focusing on lysine residues shown to be acetylated by p300, SRC-1 and SRC-3 in *in vitro* acetylation assays. Since the recruitments of SRC-1 and SRC-3 to the enhancer of the EPO gene were eliminated in cells in which p300 is knocked down, we also hypothesized that hypoxic induction of histone acetylation at the promoter of the gene would be reduced in cells with p300 knocked down if p300, SRC-1 and/or SRC-3 are responsible for these acetylations. Therefore, we performed the same analysis in Hep3B/shp300 cells. Since hypoxia (1% O_2_) treatment resulted in a greater induction of the EPO gene than DFO, we treated the cells with 1% O_2_ for these experiments, except in the case for acetylation at H4 K5, K8, K12 and/or K16, since this acetylation could also be observed at the promoter region of the EPO gene in cells treated with DFO. Since the antibody used in this last experiment was produced with a peptide corresponding to amino acids 2–19 of Tetrahymena histone H4 (AGG(Ac)KGG(Ac)KGMG(Ac)KVGA(Ac)KRHS-C), acetylated on lysines 5,8,12 and 16, as antigen, we cannot tell which specific lysine(s) is acetylated. We found that lysines at positions 23 of histone 3 (H3K23), previously shown to represent a potential *in vivo* acetylation substrate of CBP/p300 in the pS2 promoter [33], [34], was strongly acetylated after hypoxic stimulation, that this acetylation was persistent throughout the time course of hypoxia induction, and that the acetylation were eliminated in Hep3B/shp300 cells (Fig. 5A). We confirmed this conclusion by real time PCR quantization of the ChIP data at one time point (Fig. 5B). We also observed that hypoxia induced strong acetylation at lysines 5, 8, 12, and/or 16 of histone 4, which represent acetylation substrates of both p300 and SRC-1 [35], [22], in Hep3B/shscx cells but not in Hep3B/shp300 cells (Fig. 5C). Furthermore, acetylation at (one or more of) these sites was observed after DFO treatment in Hep3B/shscx but not in Hep3B/shp300 cells, and occurred in a periodic fashion (Fig. 5D). Thus p300 is required for hypoxia stimulated acetylations at these sites. It is of interest that the kinetics of acetylations of H4K5K8K12K16 at the EPO promoter is similar to the kinetics of the recruitment of p300 to the EPO enhancer, implying that H4K5K8K12 and/or K16 might be direct targets of acetylation by p300 *in vivo*. In contrast, although knocking down Brm and Brg-1 impaired the hypoxia induced association of pol II with the promoter (Fig. 4D), it did not inhibit the acetylations at H4K5K8K12K16 (Fig. 5E), indicating that Brm/Brg-1 is not required for histone modifications at the promoter, as least for acetylation at H4K5K8K12K16.

### The coactivators exhibit highly specificities towards HIF target genes

**Figure 6:**
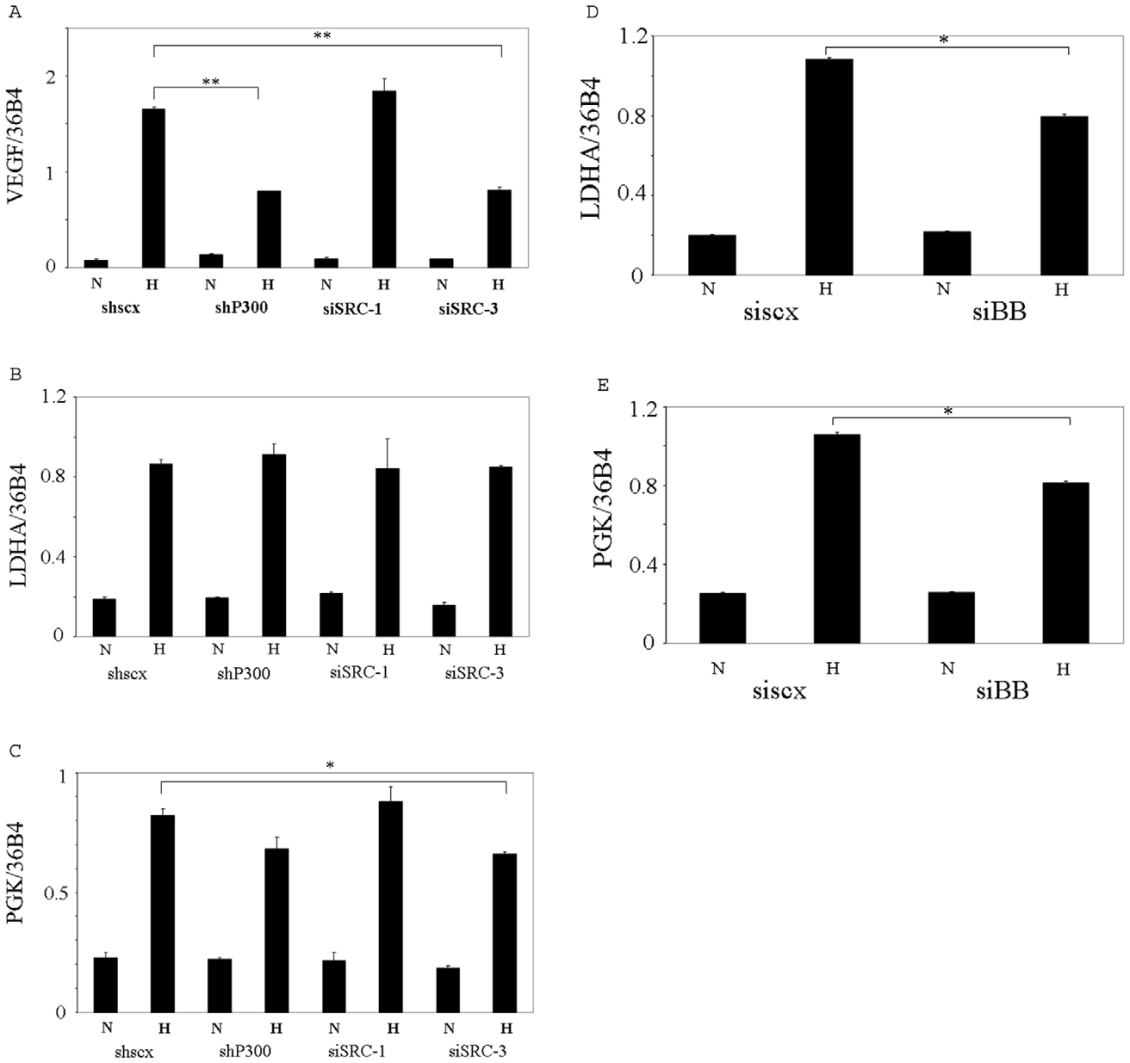
Roles of coactivators in hypoxia induced transcription of HIF-1α target genes. A, B and C, the hypoxia induced transcription of VEGF, PGK and LDHA genes were examined as described in Fig. 1B. D and E, Hep3B were transfected with siscx or siB/B. 72 h after transfection, the cells were treated with 1% O_2_ for 24 h. Then total RNA were isolated and subjected to reverse transcription and real-time PCR for analysis of VEGF, LDHA and PGK mRNA expression. N: normoxia. H: hypoxia. ** indicates statistically significant difference (p<0.01). * indicates statistically significant difference (p<0.05).

**Figure 7:**
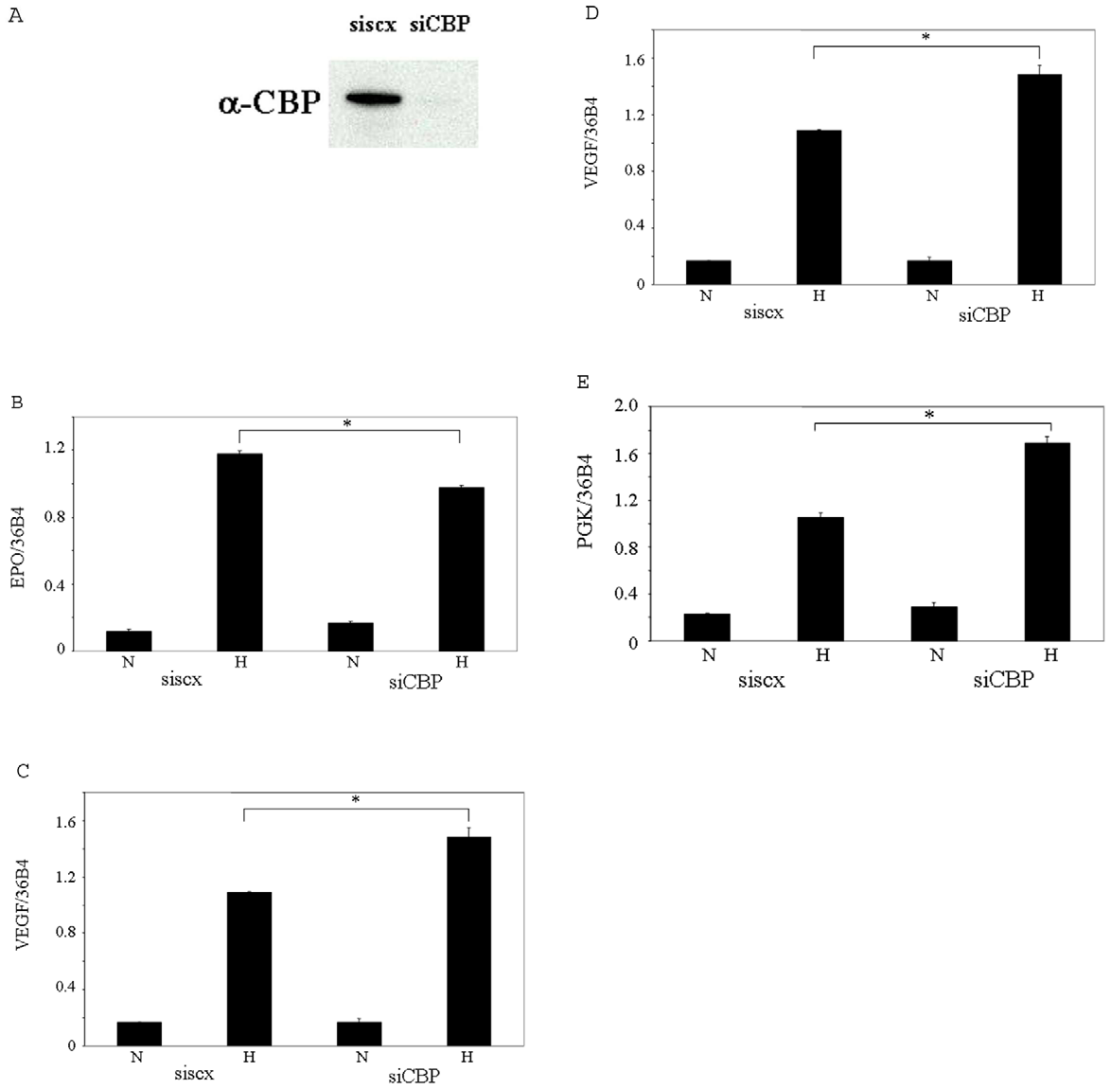
Role of CBP in hypoxia induced transcription of HIF target genes. A, whole cell extracts were prepared from Hep3B cells transfected with siscx or siCBP RNA duplexes for 72 h and then subjected to Western blot analysis using an antibody directed to CBP. B, C, D and E, total RNA was extracted from Hep3B cells transfected with siscx or siCBP RNA duplexes for 72 h and then subjected to reverse transcription and real-time PCR using primers for the EPO, VEGF, PGK and LDHA genes. N: normoxia. H: hypoxia. * indicates statistically significant difference (p<0.05).

CBP/p300 and the p160 coactivators are known to interact with the transactivation domain of HIF-1α *in vitro*. However, their roles in transactivation of endogenous target genes of HIF are unclear. We therefore examined hypoxia induction of a number of HIF target genes in Hep3B cells in which these coactivators were knocked down individually. We found that p300 and SRC-3, but not SRC-1, are required for the hypoxia induced expression of the VEGF gene, a target gene of HIF-1α in Hep3B cells (Fig. 6A), which is thus similar to EPO with regard to the requirement of coactivitors for its transcription, although EPO is uniquely a HIF-2α target in these cells. All these coactivators appeared to be dispensible for hypoxia-induced transcription of the HIF-1α target gene, LDHA, while SRC-3 appeared to be required for hypoxia induced transcription of the HIF-1α target gene, PGK (Fig. 6B and C). We confirmed these results using siRNAs with different target sequences (Fig. S1 of Electronic Supplementary Information). We previously showed that Brm and Brg-1 are required for hypoxia induced transcription of the EPO gene but not for that of the VEGF gene in Hep3B cells [26]. In the current study, we found that Brg-1 and/or Brahma appear to be required for hypoxia induced transcription of both the LDHA and PGK genes (Fig. 6D and E). Finally, we investigated the role of the p300 homolog, CBP, in hypoxia induced transcription of the genes. Knocking down CBP in Hep3B cells by siRNA (Fig. 7A) significantly decreased the hypoxia induced transcription of the EPO gene (although to a less extent than knocking down p300), suggesting that both p300 and CBP are required for hypoxia induction of EPO and that neither can completely compensate for the function of the other (Fig. 7B). Surprisingly, knocking down CBP enhanced the hypoxic induction of the VEGF, PGK and LDHA genes in Hep3B cells (Fig. 7C, D and E), suggesting that CBP and p300 are not functionally interchangeable in transactivation of the VEGF gene and that p300 and CBP are either dispensable or redundant in the transcription of the LDHA and PGK genes. The enhancement of these genes' transcription upon knocking down CBP, we speculate, results from the recruitment of other coactivators even more potent than CBP. Of interest, we observed a similar enhancement of EPO transcription by depletion of endogenous Brg-1 in our previous study [26]. Taken together, these data indicate that the roles of these coactivators in transcription are highly gene-specific even for the transcription of the genes mediated by the same transcriptional factor, either HIF-1α or HIF-2α.

## Discussion

In mammalian systems, the EPO gene is the most hypoxia-responsive gene identified, being inducible by hypoxia up to 100-fold. Transcriptional regulation is achieved by the concerted action of several transacting factors interacting with the 5′ proximal promoter region and with the 3′ untranslated region of the EPO gene, which are widely separated from each other. Hypoxic regulation of HIF activity is partially mediated by the hydroxylation of an asparagine residue in the C-terminus of HIF-1α and HIF-2α by the O_2_-dependent FIH (factor inhibiting HIF). Hydroxylation of the asparagine residue has been shown to prevent interaction of p300 with HIF-1α *in vitro* [4]. p300 has also been shown to be required for the hypoxic induction of a reporter gene driven by the EPO enhancer, to form a complex with the HRE and HIF-1α under hypoxic conditions in a gel shift assay, and to be required for hypoxia induction of endogenous VEGF [14], [15], [16]. However, information about how p300 regulates endogenous HIF target genes is still very limited. In particular, we addressed the following issues. (i) Are all or only some of the members of the p160 family involved? (ii) How do the coactivators cooperate with each other to regulate hypoxia induction of the EPO gene? (iii) What covalent chromatin modifications occur at the promoter region of the EPO gene in response to hypoxia treatment? (iv) What are the roles of p300 and the p160 family of coactivators in chromatin modification at the promoter? (v) What are the roles of these coactivators in transcriptional regulation of different endogenous HIF-1α and HIF-2α target genes? (vi) Both CBP and its homolog, p300 have been shown to bind to the C-terminal of HIF-1α and potentiate the transcriptional activities of both HIF-1α and HIF-2α in transient reporter assays. Little is known about potential differences between CBP and p300 in potentiating the transcriptional activities of HIF-1α and HIF-2α. Are they interchangeable in potentiating hypoxia induced transcription of the EPO gene and other HIF target genes? The results of our studies establish a central coactivator role for p300 in hypoxia induction of EPO.

Depletion of p300 or SRC-3 resulted in a significant reduction in transcription of the endogenous EPO gene triggered by hypoxia. To our surprise, depletion of neither SRC-1 nor SRC-2 affected the hypoxia induction of the EPO gene, although both coactivators have been shown to potentiate the transcriptional activity of HIF-1α in transient transfection assays. Thus, neither SRC-1 nor SRC-2 appears to be required for hypoxic stimulation of the EPO gene or alternatively, these p160 coactivators function redundantly in transactivation of the gene. RNA polymerase II (pol II) persistently associated with the promoter throughout the time course of hypoxic treatment, and this association was eliminated by knocking down p300, indicating that p300 is important for the initiation of the transcription of the gene. p300, SRC-1 and SRC-3 were recruited to the 3′ enhancer region of the EPO gene in response to hypoxic stimulation, bound to the enhancer as soon as 25 minutes after treatment with the hypoxia mimic, desferrioxamine, and subsequently associated with the enhancer in a periodic fashion. Cyclic pattern of recruitments of coactivators to the promoters of estrogen responsive genes, such as the cathepsin D (CATD) and pS2 genes, are well documented, and appear to reflect the association of different protein complexes of coactivators and/or corepressors with transcription factors in a sequential, combinatorial or parallel fashion [29], [30]. Recruitment of both SRC-1 and SRC-3 was found to be dependent on p300, which suggests a central coactivator role for p300. Intriguingly, SRC-1 and SRC-3 bound to the EPO enhancer with different cyclic patterns from p300. We speculate that p300 is required for the binding of SRC-1 and SRC-3 to the enhancer in the first round of recruitment after DFO treatment, but is no longer required for the subsequent rounds of recruitment, due to changes in the contexts of the enhancer and promoter of the gene, such as the histone modifications occurring at the promoter region and the physical contact between the enhancer and promoter via DNA looping. We previously showed that Brm and Brg-1 are recruited to the enhancer of the EPO gene after hypoxia treatment and are required for hypoxia induction of the gene. In the current study, we showed that depletion of p300 did not significantly impair the recruitment of Brm/Brg-1 to the enhancer, suggesting that p300 and Brm/Brg-1 belong to different protein complexes. To further investigate the relationship between p300 and Brm/Brg-1, we assessed hypoxia induced expression of EPO in cells knocked down for p300 and Brm/Brg-1 simultaneously, and observed that the effects of knocking down these coactivators was additive, supporting our notion that p300 and Brm/Brg-1 belong to different protein compelexes.

We then studied histone modifications at the promoter region, focusing on lysine residues previously identified as substrates of p300, SRC-1 or SRC-3 by other investigators using *in vitro* acetylation assays. We found that lysine at position 23 of histone 3 (H3K23) was acetylated after hypoxic stimulation. We also observed that hypoxia induced strong acetylation at lysines 5, 8, 12, and/or 16 of histone 4 and that this acetylation occurred in a cyclic fashion upon treatment with DFO, suggesting that deacetylases may be recruited to the promoter non-synchronously with HATs during regulation of EPO expression. We further investigated the role of p300 in the histone modifications and observed that the hypoxia-stimulated acetylations at K23 of H3 and K5K8K12K16 of H4 at the promoter were eliminated in cells depleted for p300, suggesting that they are bona fide substrates of p300 or p300-bound HATs.

We also investigated the roles of p300 and p160 coactivators in hypoxia induced transcription of the endogenous HIF-1α target genes VEGF, PGK and LDHA, in addition to the HIF-2α target gene, EPO. Surprisingly, while p300 was required for hypoxia induction of EPO and VEGF, it appeared to be dispensable for induction of PGK and LDHA. SRC-3 was required for hypoxia induction of PGK and VEGF but not for that of LDHA, while SRC-1 appeared not to be required for the hypoxia induced transcription of any of the genes that we examined in this study, suggesting that SRC-1 is either redundant or dispensable in transactivation of these genes. These data suggests that coactivators may play different roles even in transcriptional regulation of genes mediated by the same transcription factor. Although Brm and Brg-1 are not required for transactivation of VEGF [26], they appeared to be required for hypoxia induction of LDHA and PGK, suggesting that the promoters of both of these latter genes maintain compacted nucleosomal configurations which need to be remodeled by the SWI/SNF protein complex. SRC-1 was previously shown to enhance the transactivation potential of HIF-1α [14], [15], which might appear to be in contradiction to our observations. This discrepancy could be explained by the fact that the previous reports investigated the function of SRC-1 by overexpressing it together with a reporter gene driven by the EPO enhancer and a cDNA for HIF-1α, while we studied the expression of endogenous HIF-responsive genes via siRNA-mediated knock down. Overexpressed proteins can exhibit artificial effects, and studies of transcriptional regulation using reporter genes may lead to loss of target gene specificity.

CBP is a homolog of p300. The high degree of homology between CBP and p300 suggests that these proteins could, at least in part, be functionally redundant, and they are frequently found to be interchangeable when transiently overexpressed in cells [36]. However, accumulating evidence indicates that CBP and p300 also have unique roles *in vivo* [31]. Both proteins have been shown to bind the C-terminals of HIF-1α and HIF-2α and potentiate their transcriptional activities [4], [14], [15], [16], [17]. Hence, it is of interest to study whether/how they function differentially in hypoxia induced transcription of endogenous HIF target genes. Knocking down CBP by siRNA marginally, but significantly decreased the hypoxia induced transcription of the EPO gene, suggesting that both p300 and CBP are required for hypoxia induction of EPO, and that neither can completely compensate for the function of the other in transcriptional regulation of this gene. Surprisingly, knocking down CBP resulted in enhancement of expression of other HIF-1α regulated genes, including VEGF, PGK and LDHA. These data suggest that CBP and p300 play different roles in transactivation of the VEGF gene and they may be redundant or dispensable in transactivation of the LDHA and PGK genes.

It should be noted that in the adult organism the majority of EPO protein is produced by the renal interstitial cells where an element lying a considerable distance 5′-upstream (between −9.5 and −14 kb) of the gene directs its expression [37], [38]. Further studies of EPO regulation in renal interstitial cells will be important, given that these cells are of medical relevance in adults.

While HIF-1α was the first HIF-α subunit to be identified and has been most extensively studied, recent examination of HIF-2α in the context of both development and tumorigenesis demonstrates significant differences in the biological function of these two proteins [39]. Therefore, it will be of interest to investigate how HIF-1α and HIF-2α exert these different physiological effects. One possible mechanism underlying their different roles in physiological processes is that they regulate different hypoxia inducible genes [39].

Taken together, our studies further illuminate the roles that coactivators play in transcriptional regulation of EPO and other HIF target genes and establish a central coactivator role of p300 in hypoxia-induced transcription of the EPO gene.

## Supporting Information

Figure S1 Effect of knocking down p300, SRC-1 and SRC-3 with additional siRNAs on hypoxic induction of EPO, VEGF, LDHA and PGK. A, Hep3B cells were transfected with sip300′, siSRC-1′, siSRC-3′ and the scrambled RNA duplex, used as control. The sense sequences of sip300′, siSRC-1′, siSRC-3′ and SCX, a scrambled RNA oligonucleotide, are r(GAAAUUAGGUUACACAACAUU), r(CAGCGGGAACUGUACAGUCAA)d(TT), r(AAGGUUGUCAAUAUAGAUACA)d(TT) and r(UUCUCCGAACGUGUCACGU)d(TT). The cells were harvested and the whole cells extracts were prepared 72 h after transfection. Western blot were done using the whole cells extracts and the antibodies as indicated. B, C, D and E, Hep3B cells were transfected with siRNAs for 72 h. During the last 24 h the cells were treated with 1% O_2._ Total RNA were isolated and subjected to reverse transcription and real-time PCR. Each of the real-time PCRs was done three times and one representative result is presented. This result represent an average from three real-time PCR reactions with the same template. These genes mRNAs were normalized to that of the constitutively expressed 36B4 gene encoding a ribosomal subunit. * indicates statistically significant difference from the cells transfected with the non-targeting sequence (siscx) (p<0.01).
(0.56 MB TIF)
