# Supplementary figures and images for: Roles of Coactivators in Hypoxic Induction of the Erythropoietin Gene
Source: PLoS One. 2010 Apr 2;5(4):e10002. doi: 10.1371/journal.pone.0010002 (PMC2848849; doi:10.1371/journal.pone.0010002)

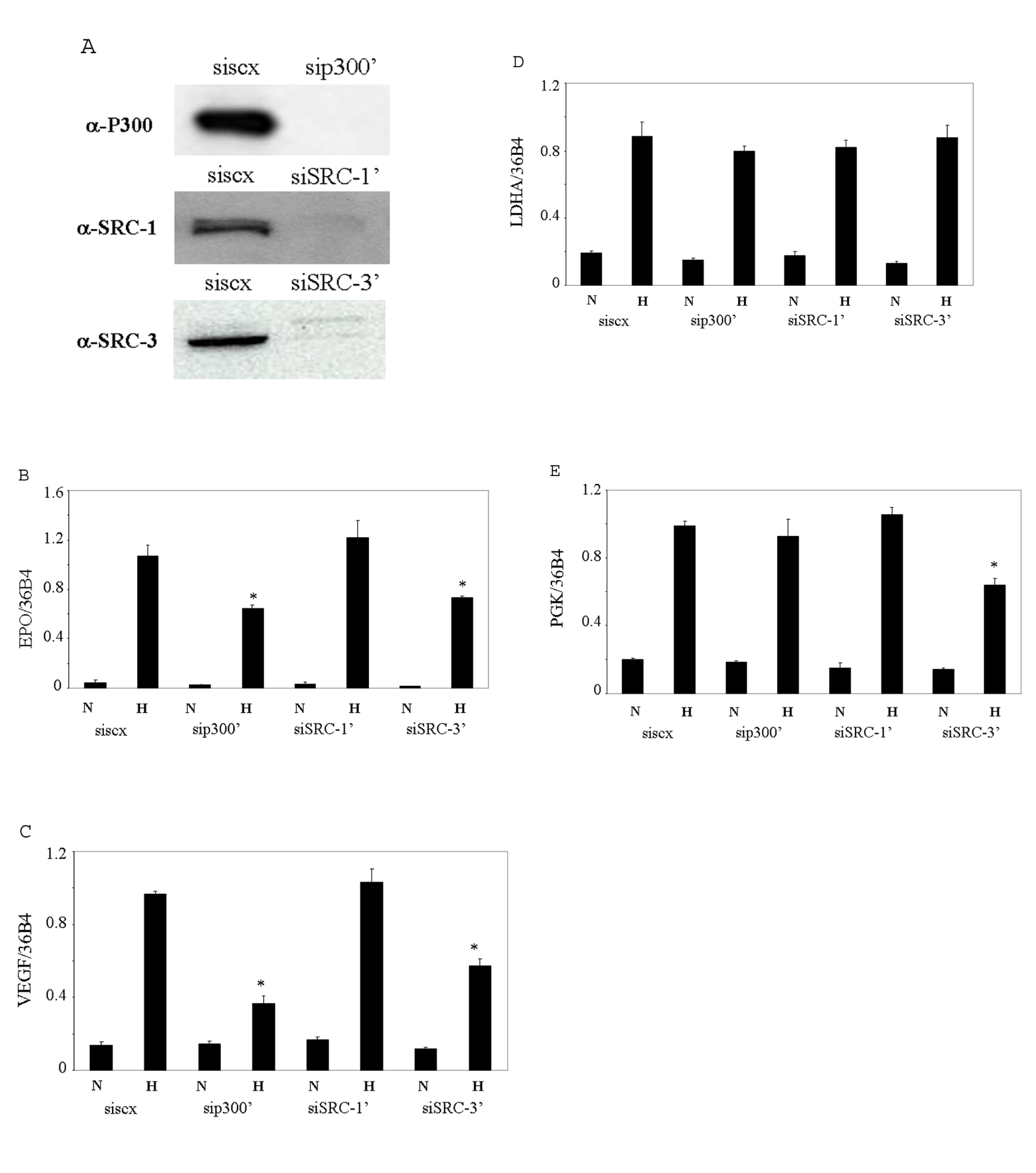

Supplement: Figure S1 — Effect of knocking down p300, SRC-1 and SRC-3 with additional siRNAs on hypoxic induction of EPO, VEGF, LDHA and PGK. A, Hep3B cells were transfected with sip300′, siSRC-1′, siSRC-3′ and the scrambled RNA duplex, used as control. The sense sequences of sip300′, siSRC-1′, siSRC-3′ and SCX, a scrambled RNA oligonucleotide, are r(GAAAUUAGGUUACACAACAUU), r(CAGCGGGAACUGUACAGUCAA)d(TT), r(AAGGUUGUCAAUAUAGAUACA)d(TT) and r(UUCUCCGAACGUGUCACGU)d(TT). The cells were harvested and the whole cells extracts were prepared 72 h after transfection. Western blot were done using the whole cells extracts and the antibodies as indicated. B, C, D and E, Hep3B cells were transfected with siRNAs for 72 h. During the last 24 h the cells were treated with 1% O2. Total RNA were isolated and subjected to reverse transcription and real-time PCR. Each of the real-time PCRs was done three times and one representative result is presented. This result represent an average from three real-time PCR reactions with the same template. These genes mRNAs were normalized to that of the constitutively expressed 36B4 gene encoding a ribosomal subunit. * indicates statistically significant difference from the cells transfected with the non-targeting sequence (siscx) (p<0.01). (0.56 MB TIF) [file pone.0010002.s001.tif]
